# Using Genes as Characters and a Parsimony Analysis to Explore the Phylogenetic Position of Turtles

**DOI:** 10.1371/journal.pone.0079348

**Published:** 2013-11-21

**Authors:** Bin Lu, Weizhao Yang, Qiang Dai, Jinzhong Fu

**Affiliations:** 1 Chengdu Institute of Biology, Chinese Academy of Sciences, Chengdu, Sichuan, China; 2 Department of Integrative Biology, University of Guelph, Guelph, Ontario, Canada; Sars International Centre for Marine Molecular Biology, Norway

## Abstract

The phylogenetic position of turtles within the vertebrate tree of life remains controversial. Conflicting conclusions from different studies are likely a consequence of systematic error in the tree construction process, rather than random error from small amounts of data. Using genomic data, we evaluate the phylogenetic position of turtles with both conventional concatenated data analysis and a “genes as characters” approach. Two datasets were constructed, one with seven species (human, opossum, zebra finch, chicken, green anole, Chinese pond turtle, and western clawed frog) and 4584 orthologous genes, and the second with four additional species (soft-shelled turtle, Nile crocodile, royal python, and tuatara) but only 1638 genes. Our concatenated data analysis strongly supported turtle as the sister-group to archosaurs (the archosaur hypothesis), similar to several recent genomic data based studies using similar methods. When using genes as characters and gene trees as character-state trees with equal weighting for each gene, however, our parsimony analysis suggested that turtles are possibly sister-group to diapsids, archosaurs, or lepidosaurs. None of these resolutions were strongly supported by bootstraps. Furthermore, our incongruence analysis clearly demonstrated that there is a large amount of inconsistency among genes and most of the conflict relates to the placement of turtles. We conclude that the uncertain placement of turtles is a reflection of the true state of nature. Concatenated data analysis of large and heterogeneous datasets likely suffers from systematic error and over-estimates of confidence as a consequence of a large number of characters. Using genes as characters offers an alternative for phylogenomic analysis. It has potential to reduce systematic error, such as data heterogeneity and long-branch attraction, and it can also avoid problems associated with computation time and model selection. Finally, treating genes as characters provides a convenient method for examining gene and genome evolution.

## Introduction

**Figure 1:**
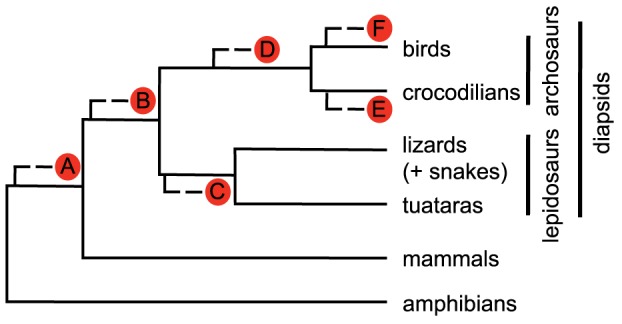
Alternative placements of turtles in the current phylogeny of living tetrapods.

Despite numerous recent attempts, the phylogenetic position of turtles remains controversial [1]–[6]. Traditionally, turtles were placed as the sister-group to A) all other living amniotes [7], or B) diapsids, but with various affiliations to different extinct groups [8]–[11] (see [12] for a review). These views are primarily based on morphological and fossil data. Most recent studies, however, favor a more nested position for turtles within diapsids. In addition to morphological data [13], [14], molecular data overwhelmingly support this placement. Several potential positions have been proposed, including turtles being the sister-group to C) lepidosaurs [2], [15], D) archosaurs [5], [6], [16]–[20], E) crocodilians [1], [21]–[23], and F) birds [24]. The alternative placements of turtles in the current phylogeny of living tetrapods are shown in Figure 1.

Conflicting conclusions are likely a consequence of systematic error rather than sampling error in the tree construction process, because most recent studies utilized substantial amounts of data. For example, Tzika et al. used transcriptome data of 4,689 genes [1], Lyson et al. used present/absent data of 186 microRNA families [2], and Wang et al. used 1,113 coding genes [6]. Conventional sequence data analysis uses nucleotide sites (or amino-acid residues) as characters. When data from a large number of genes are available, a supermatrix approach is often preferentially used (concatenated data analysis; e.g. [25]–[28]). Several well-recognized systematic errors are associated with this approach. Data heterogeneity is probably the most pronounced error in large dataset analysis, such as in phylogenomics [29], [30]. Additionally, when divergence is deep, multiple ‘hits’ in DNA substitution often lead to long-branch attraction [1], [31]. Furthermore, with a large and heterogeneous dataset, a realistic substitution model becomes elusive. Fitting different parts of the data with different sets of parameters may over-parameterize the model [32]. Several recent studies suggested that conventional concatenated sequence analysis might be problematic for phylogenetics with large amounts of data [30], [33].

**Figure 2:**
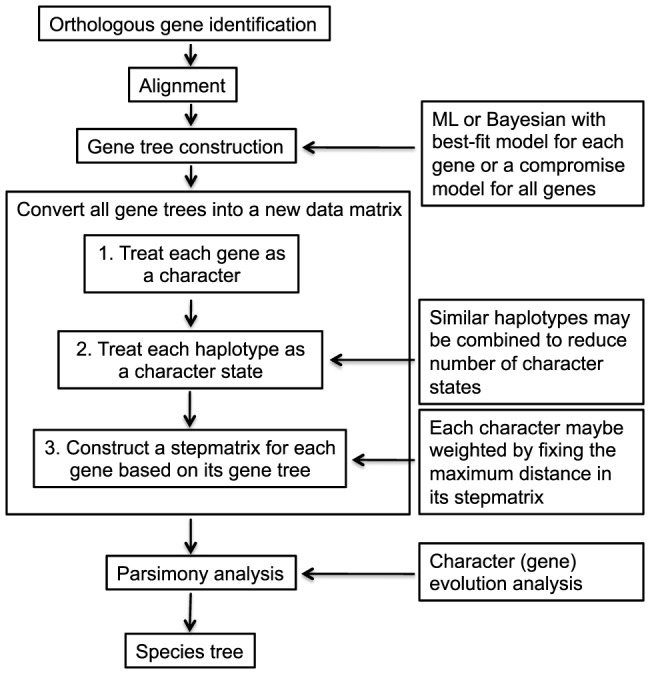
The procedural flowchart of using genes as characters in phylogenomic analysis.

A potentially better alternative in the age of phylogenomics is to use genes as characters, haplotypes as character states, and gene trees as character-state trees. Each gene represents one ordered multi-state character, and parsimony principle can be applied to reconstruct the species phylogeny (Figure 2). The idea of “locus (gene) as character” was first discussed in the isozyme community [34]. Doyle (1992) further explicitly proposed using a gene tree as one single species tree character [35]. Conceptually, genes are the unit of inheritance and function in Mendelian genetics, and arguably are a better choice than nucleotide sites (or amino-acid residues) as characters in evolutionary biology. Theoretically, this approach is capable of mitigating systematic error associated with supermatrix analysis using nucleotide sites as characters (e.g., data heterogeneity). The recent development of DNA sequencing technology makes massive amounts of sequence data readily available, which in turn makes the genes as characters approach a practical alternative. With the rapid development of phylogenomics, advantages associated with the genes as characters approach should be explored.

In this study, we evaluate the phylogenetic position of turtles within the vertebrate tree of life using the genes as characters approach with genomic data. We first sequenced a transcriptome of a turtle species and retrieved genomic data of ten other amniotes from other sources. Both concatenated data analysis and genes as characters approach were then applied and compared. Finally, utilization of phylogenetic information to understand genome evolution, including gene modular evolution and gene function association, was explored.

## Results

### Data acquisition

We first acquired high quality transcriptome data of a turtle species (Chinese pond turtle, *Mauremys reevesii*) by RNA sequencing. A total of 35,399,774 reads were generated through Illumina sequencing. After *de novo* assembly, 61,810 transcripts were produced with an N50 length of 1,571 base pairs (bps) and an average length of 1,072 bps. Sequence lengths ranged from 295 bps to 19,374 bps. Sequence information and the length distribution of transcripts are presented in Table S1 and Figure S1. Transcriptomic or genomic data of another ten vertebrate species were acquired from online databases, including green anole (*Anolis carolinensis*), Nile crocodile (*Crocodylus niloticus*), chicken (*Gallus gallus*), human (*Homo sapiens*), opossum (*Monodelphis domestica*), soft-shelled turtle (*Pelodiscus sinensis*), royal python (*Python regius*), zebra finch (*Taeniopygia guttata*), tuatara (*Sphenodon punctatus*), and western clawed frog (*Xenopus tropicalis*).

Putative orthologous genes were identified using the program HaMSTR (Hidden Markov Model based Search for Orthologs using Reciprocity; [36]) and the Amniota Orthology Dataset. Based on the six species in the Amniota Orthology Dataset, 5,587 putatively orthologous proteins and 5,584 coding genes of *M. reevesii* were initially identified. After removing the dubious orthologs based on the individual gene tree (see below), 4,584 genes remained for both amino-acid and nucleotide data for these seven species. When we added the transcriptome data of the other four species, *Pelodiscus sinensis*, *Crocodylus niloticus*, *Python regius*, and *Sphenodon punctatus*, the number of orthologous genes sharply decreased to 1,638 in the final amino-acid and nucleotide data. Considering the levels of details of the acquired data, we constructed two datasets for phylogenetic analysis. Seven species (human, opossum, zebra finch, chicken, green anole, Chinese pond turtle, and western clawed frog) had many more orthologous genes than the other species, and formed a 7-species dataset. The second dataset, the 11-species dataset, had more taxa but fewer genes. The transcriptome raw data of *M. reevesii* are deposited at the NCBI Sequence Read Archive (accession number SRA098741), and the putative orthologous gene sequence data of *M. reevesii* are deposited at GenBank (accession number GANJ01000000).

### Phylogenetic inferences

Phylogenetic trees were constructed based on both amino-acid and coding region nucleotide sequences. Alignments were conducted for each individual gene. The western clawed frog served as outgroup in all phylogenetic analyses.

We first analyzed each gene separately to obtain gene trees using maximum likelihood (ML) method. We noticed that some gene trees produced by nucleotide sequences had unusually long branches, and careful inspection of the data suggested that low quality alignments, non-orthologous genes, or potential assembling errors might have contributed to the long branches. Consequently, gene trees with the longest branch length greater than 1.5, which included 1004 genes for the 7-species dataset and 642 genes for the 11-species dataset, were excluded from subsequent analyses.

#### Concatenated data analysis

**Figure 3:**
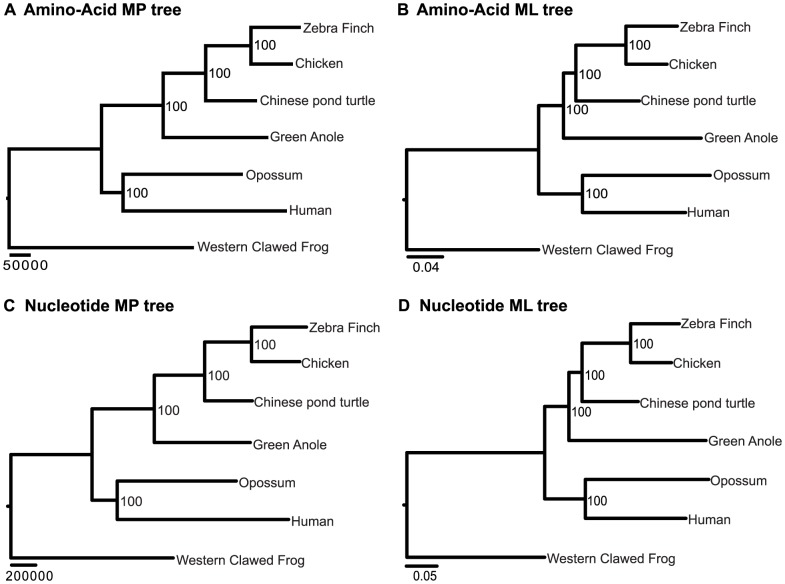
The phylogenetic hypotheses derived from the 7-species data. Amino-acid and nucleotide sequences were analyzed by maximum parsimony (MP) and maximum likelihood (ML) methods, respectively. Numbers near the nodes are bootstrap values.

All amino-acid and nucleotide sequences were pooled together, respectively. The data were analyzed using maximum parsimony (MP), ML, and Bayesian inference methods. The concatenated 7-species amino-acid dataset included 2,630,903 amino-acid residuals. Both the ML and MP tree strongly supported turtles being the sister-group to archosaurs (the archosaur hypothesis; Figure 3 A–B). The concatenated nucleotide sequence was 7,843,295 bp long. For parsimony analysis, due to computation constraints, the dataset was reduced to 3,824,484 bps by removing all constant sites. The ML and MP analyses produced the same topology as that of amino-acid sequences (Figure 3 C–D).

**Figure 4:**
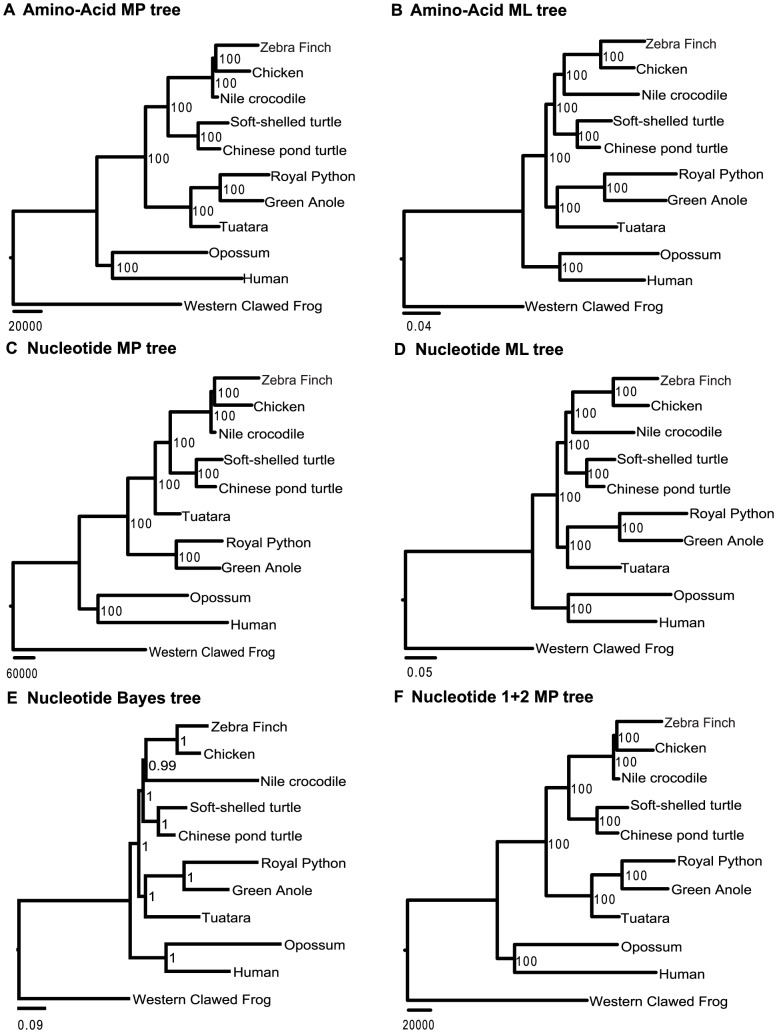
The phylogenetic hypotheses derived from the 11-species data. Amino-acid and nucleotide sequences were analyzed by maximum parsimony (MP) and maximum likelihood (ML) methods, respectively. A Bayesian tree from nucleotide sequences and a MP tree from the 1^st^ and 2^nd^ codon position sequences are also presented. Numbers near the nodes are bootstrap values or Bayesian posterior probabilities (E).

The concatenated 11-species amino-acid dataset included 808,806 amino-acid residuals, and both the ML and MP trees again placed turtles as sister-group to archosaurs (Figure 4 A–B). The concatenated nucleotide sequences included 2,431,766 nucleotide sites. All ML, MP, and Bayesian trees supported turtles being the sister-group to archosaurs (Figure 4 C–E). They had however, different placement for tuatara; while ML and Bayesian trees placed tuatara as the sister-group to squamates ( = lizards+snakes), MP tree placed tuatara as the basal clade to the (archodaurs+turtles) clade (Figure 4 C). Removing the hyper-variable 3^rd^ codon position, the MP analysis resulted in tuatara being the sister-group to squamates (Figure 4 F). In all cases of concatenated analysis, all nodes received high bootstrap support ( = 100) or Bayesian posterior probability support ( = 99 or 100).

#### Genes as characters method

We used two methods to summarize the information from gene trees into species trees. First, we used a simple count method, similar to the gene-support frequency measurement [30]. The numbers of genes that support various hypotheses were counted and compared. The hypothesis supported by the highest number of genes was preferred. Second, we performed a more elaborated parsimony analysis following the arguments of Doyle [35]. Each gene was treated as a single ordered multi-state character. Haplotypes of a gene were treated as character states and the gene trees were treated as the character-state trees. Step matrices were used to describe the character-state trees. We weighted all characters (genes) equally. The maximum pairwise distance in each step matrix was set to ten and all other steps were proportionally scaled. Finally, a parsimony analysis was performed. The procedure is summarized in Figure 2.

For the 7-species data, 4,584 individual proteins (∼574 amino-acids per protein) and coding genes (∼1,711 base pair per gene) were analyzed to reconstruct gene trees. Among them, 1,868 proteins (41%) and 2,117 coding genes (46%) supported the archosaur hypothesis. In contrast, only 811 proteins (18%) and 798 coding genes (17%) supported the lepidosaur hypothesis. Surprisingly, there were 535 (12%) proteins and 460 (10%) coding genes that supported the diapsid hypothesis (Table 1). For the 11-species data, 1,638 individual proteins and coding genes were analyzed to reconstruct gene trees. Among them, 80 proteins (5%) and 222 coding genes (14%) supported the archosaur hypothesis, 80 proteins (5%) and 121 coding genes (7%) supported the lepidosaur hypothesis, 22 proteins (1%) and 90 coding genes (5%) supported the crocodilian hypothesis, and 17 proteins (1%) and 47 coding genes (3%) supported the bird hypothesis (Table 1). Generally, the archosaur hypothesis received support from the largest number of genes. Noticeably, however, both the archosaur hypothesis and the lepidosaur hypothesis received support from the same number of proteins in the 11-species data. The diapsid hypothesis also received substantial support from a large number of genes (10–12%). In addition, amino-acid sequences were less informative than nucleotide sequences, and a larger portion of the gene trees from amino-acid sequences included polytomies.

**Table 1:** A simple tally of genes that support alternative hypotheses.

Hypothesis	Tree topology	Number of genes (nucleotides)	Number of proteins (amino-acid)
**7-species dataset**			
Archosaur Hypothesis	((ZF,Ch),PT)	2117 (46%)	1868 (41%)
	(((ZF,Ch),PT)An)	1502	1327
	(((((ZF,Ch),PT),An),(Hu,Op)),Fr)	1281	1149
Lepidosaur Hypothesis	(PT,An)	798 (17%)	811 (18%)
	((PT,An),(ZF,Ch))	618	597
	((((PT,An),(ZF,Ch)),(Hu,Op)),Fr)	500	501
Diapsid Hypothesis	((ZF,Ch,An),PT)	460 (10%)	535 (12%)
	(((((ZF,Ch),An),PT),(Hu,Op)),Fr)	379	450
Other topologies		1209 (26%)	1370 (30%)
**11-species dataset**			
Archosaur Hypothesis	(((ZF,Ch),Cr),(PT,ST))	222 (14%)	80 (5%)
	((((ZF,Ch),Cr),(PT,ST)),((An,Py),Tu))	106	32
Lepidosaur Hypothesis	(((An,Py),Tu),(PT,ST))	121 (7%)	80 (5%)
	((((An,Py),Tu),(PT,ST)),((ZF,Ch),Cr))	32	21
Crocodilian Hypothesis	((Cr,(PT,ST)),(ZF,Ch))	90 (5%)	22 (1%)
Bird Hypothesis	(((ZF,Ch),(PT,ST)),Cr)	47 (3%)	17 (1%)
Other topologies		1158 (71%)	1439 (88%)

Hu = Human (*Homo sapiens*), Fr = Western Clawed Frog (*Xenopus tropicalis*), ZF = Zebra Finch (*Taeniopygia guttata*), Ch = Chicken (*Gallus gallus*), An = Green Anole (*Anolis carolinensis*), PT = Chinese Pond Turtle (*Mauremys reevesii*), ST = Soft-shelled Turtle (*Pelodiscus sinensis*), Py = Royal Python (*Python regius*), Tu = Tuatara (*Sphenodon punctatus*), Op = Opossum (*Monodelphis domestica*), Cr = Nile Crocodile (*Crocodylus niloticus*).

For example, the clade ((ZF,Ch),PT) appears on 2117 gene trees without regarding other relationships. The 7-species dataset includes 4,584 putatively orthologous proteins and coding genes; the 11-species dataset includes 1,638 putatively orthologous proteins and coding genes.

**Figure 5:**
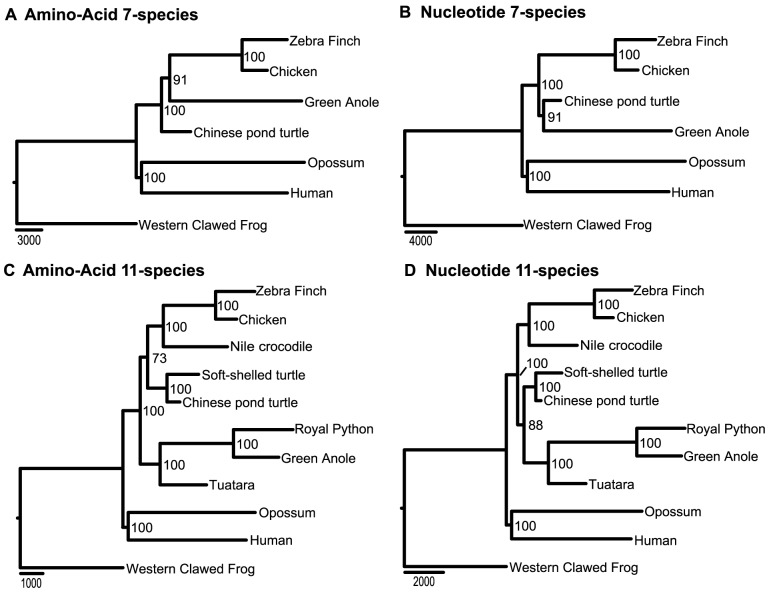
The phylogenetic hypotheses derived from using genes as characters. Individual gene tree was first estimated and used as character-state tree. Each gene was then treated as a character and a parsimony analysis was used to construct the species trees. Amino-acid and nucleotide sequences were analyzed separately. Numbers near the nodes are bootstrap values. Note the different placements of turtles and the low bootstrap values for the associated nodes.

Using each individual gene tree as a character-state tree, parsimony analyses produced very different results. The 7-species amino-acid dataset placed turtles as sister-group to the (birds+lizards) clade ( = diapsids; Figure 5 A), while the nucleotide dataset placed turtles as sister-group to lizards ( = lepidosaurs; Figure 5 B). Furthermore, the 11-species amino-acid dataset suggested turtles being the sister-group to the (birds+crocodilians) clade ( = archosaurs; Figure 5 C), while the nucleotide dataset suggested turtles being the sister-group to the (squamates+tuatara) clade ( = lepidosaurs; Figure 5 D). Noticeably, none of the above solutions received particularly high bootstrap support; the values of related nodes varied from 73 to 91.

#### Incongruence analysis

We examined the incongruence among genes with two methods. A SplitsTree analysis [37] was used to identify where, on the species tree, most incongruence occurred among the gene trees. A Phylcon analysis [38] was used to detect different levels of support of various genes to a particular topology.

**Figure 6:**
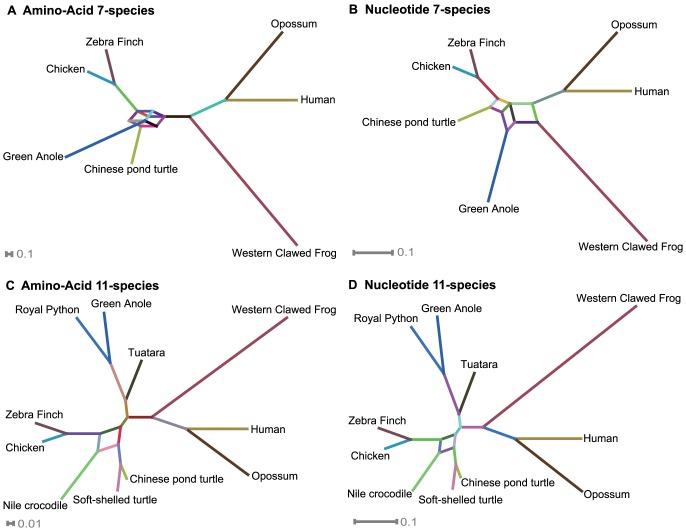
Results of the SplitTree analysis. Loops or boxes indicate the location of incongruence on the networks. The analysis examined four datasets, and all suggested strong incongruence regarding the phylogenetic position of turtles.

The SplitTree analysis clearly pointed most uncertainty to the placement of turtles in the networks (Figure 6). With the 7-species data set, the uncertainty is located at the node that includes turtle, birds, and lizards (Figure 6A), although the nucleotide data further extended the uncertainty to include mammals (Figure 6 B). For the 11-species data, the uncertainty was one node, among turtles, birds and crocodilians (Figure 6 C–D). Figure 6 presents results with a threshold value of 0.15, which most clearly demonstrates the main contradictory splits.

**Figure 7:**
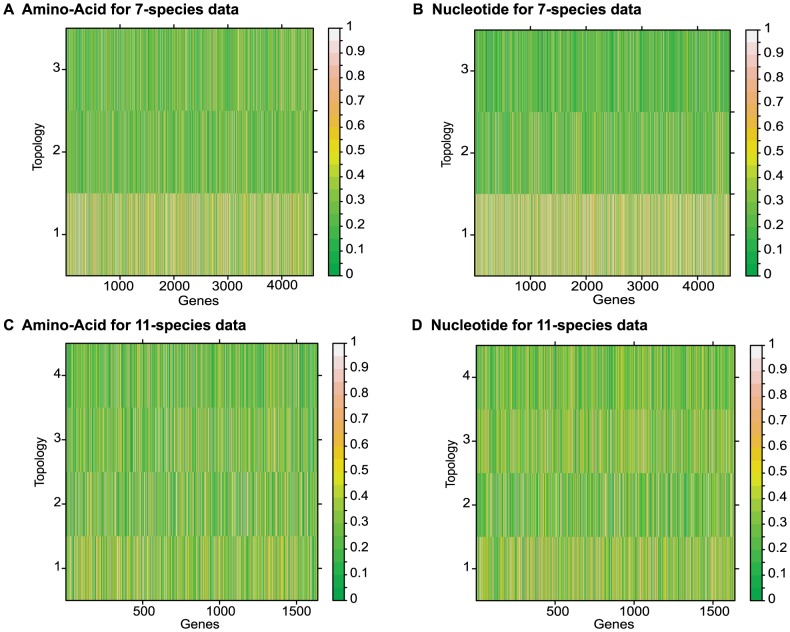
Heatmaps from the Phylcon analysis. Each vertical line represents a gene and different colors represent different p values from the AU tests. A small p value (dark green) indicates that a gene rejects a topology. Topologies examined: 7-species data: 1. (((((ZF, Ch), PT),An),(Hu, Op)), Fr); 2. ((((ZF, Ch),(PT, An)),(Hu, Op)), Fr); 3. (((((ZF, Ch),An), PT),(Hu, Op)), Fr). 11-species data: 1. (((Hu, Op),((((ZF, Ch), Cr),(PT, ST)),((An,Py),Tu))),Fr); 2. (((Hu, Op),(((ZF, Ch), Cr),((PT, ST),((An,Py),Tu)))),Fr); 3. (((Hu, Op),(((ZF, Ch),(Cr,(PT, ST))),((An,Py),Tu))),Fr); 4. (((Hu, Op),((((ZF, Ch),(PT, ST)), Cr),((An,Py),Tu))),Fr). Hu = Human (*Homo sapiens*), Fr = Western Clawed Frog (*Xenopus tropicalis*), ZF = Zebra Finch (*Taeniopygia guttata*), Ch = Chicken (*Gallus gallus*), An = Green Anole (*Anolis carolinensis*), PT = Chinese Pond Turtle (*Mauremys reevesii*), ST = Soft-shelled Turtle (*Pelodiscus sinensis*), Py = Royal Python (*Python regius*), Tu = Tuatara (*Sphenodon punctatus*), Op = Opossum (*Monodelphis domestica*), Cr = Nile Crocodile (*Crocodylus niloticus*).

The Phylcon analysis revealed a substantial amount of incongruence among genes (Figure 7). For the 7-species data, the archosaur hypothesis (H1) was clearly better supported by a larger number of genes (lighter colored genes) than the lepidosaur hypothesis (H2) and the diapsid hypothesis (H3). For the 11-species data, the archosaur hypothesis (H1) and the crocodile hypothesis (H3) were slightly better supported than the lepidosaur hypothesis (H2) and the bird hypothesis (H4). The most noticeable feature of these heatmaps, however, was the amount of conflict among genes; even for the best supported topologies by the concatenated data (the archosaur hypothesis), a large number of genes strongly rejected them (dark colored genes), particularly in the 11-species data.

### Gene evolution

Within a phylogenetic framework, we explored two aspects of gene evolution: the impact of genes experienced positive selection on phylogenetic inferences and gene modular evolution.

#### Positive selection

We used a maximum likelihood approach and the branch-site models [39] and detected signature of positive selection for 272 genes (out of 4,461) in the 7-species data and 31 genes (out of 957) in the 11-species data. We further performed a Chi-square test to examine any association between genes under selection and two primary alternative hypotheses, the archosaur and the lepidosaur hypotheses. For the 7-species dataset, 143 of the 272 genes supported the archosaur hypothesis and 37 of them support the lepidosaur hypothesis. The Chi-square test indicated that the proportion of the positively selected genes that support the archosaur hypothesis was significantly higher than expected and/or genes that support the lepidosaur hypothesis was significantly lower than expected (P = 0.0331; Table 2). Therefore, positive selection clearly had significant influence on the final tree topology.

**Table 2:** Chi-square test of the relationship between genes under positive selection and alternative hypotheses.

Numbers genes support a clade	((ZF, Ch), PT)	(PT, An)
Under positive selection	143 (130.66)	37 (49.34)
Neutral	1917 (1929.35)	741 (728.66)
Total	2060	778
Chi-square value	4.54	
P	**0.0331**	

ZF = Zebra Finch (*Taeniopygia guttata*), Ch = Chicken (*Gallus gallus*), PT = Chinese Pond Turtle (*Mauremys reevesii*), An = Green Anole (*Anolis carolinensis*).

Numbers in parentheses are expected numbers of genes under random distribution. Significantly more positively selected genes support the archosaur hypothesis.

Three genes (RUFY3, ENSG00000018189, GO: developmental protein; NUP37, ENSG00000075188, GO: Cell division, carbohydrate metabolic process and glucose transport; COG1, ENSG00000166685, GO: Golgi transport complex) were detected positive selection signal in both the 7-species and 11-species data. The function of these genes mainly involved biological regulation and cellular process. How these genes may have contributed to the evolution of turtles is worth exploring.

#### Gene function

To assess gene modular evolution, we tested whether genes supporting a particular topology were over-represented in a particular function category or metabolic pathway. The Gene Ontology (GO) annotation and the Kyoto Encyclopedia of Genes and Genomes (KEGG) were used to classify gene function. At least 22 GO categories were over-represented among genes that support the archosaur hypothesis or the lepidosaur hypothesis (Table 3). For example, 61 genes that support the archosaur hypothesis were in the functional category of ‘organophosphate metabolic process’, which was higher than expected (49 under a random distribution). Similarly, 76 genes that support the lepidosaur hypothesis were in the functional category of ‘cell activation’, which was higher than expected (61 under a random distribution). In general, it appeared that genes associated with metabolic process and binding function were likely to support the archosaur hypothesis, while genes associated with fertilization, digestion, and development were more likely to support the lepidosaur hypothesis (Table 3).

**Table 3:** Chi-square tests of the relationship between genes functions and phylogenetic hypotheses.

GO/KEGG category	A+	A−	O+	O−	X^2^	P
**A. Test of the Archosaur Hypothesis [((Zebra Finch, Chicken), Chinese Pond Turtle)]**						
**Biological Process**						
organophosphate metabolic process (GO:0019637)	61 (49)	1975 (1987)	46 (58)	2342 (2030)	5.3	0.0210
hormone metabolic process (GO:0042445)	49 (39)	1987 (1997)	35 (45)	2353 (2343)	5.2	0.0223
macromolecule metabolic process (GO:0043170)	1180 (1148)	856 (888)	1314 (1346)	1074 (1042)	3.8	0.0500
primary metabolic process (GO:0044238)	1419 (1385)	617 (651)	1590 (1624)	798 (764)	4.9	0.0269
**Cellular Component**						
organelle lumen (GO:0043233)	682 (648)	1354 (1388)	726 (760)	1662 (1628)	4.8	0.0276
extracellular matrix (GO:0031012)	77 (65)	1959 (1971)	64 (76)	2324 (2312)	4.3	0.0376
extracellular matrix part (GO:0044420)	37 (25)	1999 (2011)	18 (30)	2370 (2358)	10.1	0.0015
**Molecular Function**						
nucleotide binding (GO:0000166)	409 (381)	1627 (1655)	419 (447)	1969 (1941)	4.7	0.0307
hormone binding (GO:0042562)	25 (18)	2011 (2018)	15 (22)	2373 (2366)	4.4	0.0357
cofactor binding (GO:0048037)	72 (60)	1964 (1976)	59 (71)	2329 (2317)	4.3	0.0372
**KEGG pathway**						
inositol phosphate metabolism (map00562)	28 (18)	318 (328)	10 (20)	375 (365)	11.2	0.0008
phosphatidylinositol signaling system (map04070)	31 (22)	315 (324)	15 (24)	370 (361)	7.9	0.0049
arginine and proline metabolism (map00330)	24 (18)	322 (328)	14 (20)	371 (365)	4.0	0.0448
**B. Test of the Lepidosaur Hypothesis [(Pond Turtle, Green Anole)]**						
**Biological Process**						
cell activation (GO:0001775)	76 (61)	701 (716)	274 (289)	3373 (3358)	4.5	0.0334
translational initiation (GO:0006413)	17 (10)	760 (767)	40 (47)	3607 (3600)	6.0	0.0143
cellular component disassembly (GO:0022411)	45 (34)	732 (743)	149 (160)	3498 (3487)	4.4	0.0350
fertilization (GO:0009566)	14 (8)	763 (769)	33 (39)	3614 (3608)	4.9	0.0268
pollination (GO:0009856)	16 (10)	761 (767)	40 (46)	3607 (3601)	4.7	0.0293
system process (GO:0003008)	156 (135)	621 (642)	610 (631)	3037 (3016)	5.0	0.0250
digestion (GO:0007586)	13 (6)	764 (771)	23 (30)	3624 (3617)	8.6	0.0033
stem cell maintenance (GO:0019827)	14 (8)	763 (769)	34 (40)	3613 (3607)	4.5	0.0336
regulation of multi-organism process (GO:0043900)	15 (9)	762 (768)	34 (40)	3613 (3607)	5.8	0.0158
**Cellular Component**						
cell projection (GO:0042995)	130 (110)	647 (667)	499 (519)	3148 (3128)	4.9	0.0271
membrane part (GO:0044425)	329 (296)	448 (481)	1355 (1388)	2292 (2259)	7.3	0.0068
**Molecular Function**						
protein complex scaffold (GO:0032947)	12 (5.6)	765 (771)	20 (26)	3627 (3621)	8.8	0.0029
**KEGG pathway**						
glycerolipid metabolism (map00561)	14 (7)	119 (126)	25 (32)	573 (566)	8.7	0.0032
glycerophospholipid metabolism (map00564)	13 (7)	120 (126)	28 (34)	570 (564)	5.3	0.0210

Only significant results are presented. A: the archosaur hypothesis; L: the lepidosaur hypothesis; O: other hypotheses. +: number of genes which have a particular GO category or KEGG pathway; −: number of genes which do not have the GO category or KEGG pathway. Numbers in parentheses are expected numbers of genes under random distribution. For example, of the 2117 genes that support the Archosaur hypothesis, 2036 genes have the GO category of “biological process”; among them, 61 have the GO category of “organophosphate metabolic process” and 1975 genes do not have the term.

We detected five KEGG pathways that had biased representation. Genes involving the phosphatidylinositol signaling system, inositol phosphate and arginine and proline metabolism were more likely to support the archosaur hypothesis while genes involved with glycerolipid and glycerophospholipid metabolism were more likely to support the lepidosaur hypothesis (Table 3).

## Discussion

### Turtles as sister-group to Archosauria?

Using genes as characters, our analysis suggested that turtles are possibly the sister-group to diapsids, archosaurs, or lepidosaurs (Figure 5). Furthermore, considering the large amount of data, none of these solutions received a particularly high bootstrap support (77–91). This is clearly different from our concatenated data analysis, which supports the archosaur hypothesis with high bootstrap support ( = 100). Several recent genomic data based studies, such as Shaffer et al. [5] and Wang et al. [6], also used a concatenated approach and supported the archosaur hypothesis with high bootstrap support ( = 100). Rather than providing a clear answer, the genes as characters approach maintains the uncertainty about the phylogenetic position of turtles.

The observed level of gene incongruence suggests that the apparent solid support for the archosaur hypothesis is an artifact of concatenated analysis. Most studies supporting the archosaur hypothesis used a conventional concatenated analysis [5], [6], [19], [20], and problems associated with this approach in a phylogenomic context are well recognized [30], [33]. With a large amount of data, a putatively wrong phylogeny may be strongly supported with 100% bootstrap probability, and a topology can also be well supported even without support from any single gene [30], [33]. The high bootstrap values represent an over-estimate of confidence as consequence of large numbers of characters, and therefore, they are not appropriate measure of confidence for large concatenated datasets and should be abandoned in such cases [30]. Furthermore, our incongruence analysis clearly demonstrated that there is a large amount of inconsistency among genes and most of the conflict is surrounding the placement of turtles (Figure 6 & 7). The relatively low bootstrap values on our trees from genes as characters approach (Figure 5) are likely a reflection of the true state of nature, including conflict among gene trees and the uncertainty of the species tree. Different data treatment may favor one hypothesis over another, but they will unlikely provide any solution with statistical confidence.

Studies using other types of data and analyses reached different conclusions, and these studies cannot be discounted. For example, Lyson et al. [2] used microRNA data and supported the lepidosaur hypothesis. These data do not suffer the same problem as the concatenated sequence analysis and are proven to be reliable for phylogenetic information [40]. Hill's [12] intensive sampling of taxa, both fossil and extent, and morphological characters also resulted in turtles being the sister-group of lepidosaurs. All these conflicting results suggest that a solid conclusion of a turtle-archosaur affinity is pre-mature. This “turtle problem” is likely one of these cases that a solid phylogenetic solution is difficult, if not impossible, to achieve for several reasons, such as rapid speciation/divergence or ancient lineages, which have been extensively discussed by Delsuc et al. [41], Philippe et al. [42], and others.

### Genes as characters in phylogenomics

Using genes as characters offers an alternative to concatenated analysis for large genomic data. The debate between consensus tree versus total evidence approaches has been at the centre of modern phylogenetics for decades, and benefits of analyzing each dataset separately are well-recognized (see review [41], [43]). In the era of phylogenomics, there are additional advantages of treating each gene as an independent character. First, using genes as characters likely reduces systematic error. With the massive amount of data, random error in phylogenomics becomes smaller, but systematic error, such as data heterogeneity, is often amplified [29], [41], [44]. Both theoretical exploration and empirical case studies have demonstrated that concatenated analysis may produce misleading results with high bootstrap supports [30], [33], and analyzing genes separately is capable of reducing these errors [29], [30], [44]. Using genes as characters will also reduce the long-branch attraction problem. The ultimate cause of the problem is that there are only four alternative states (A, C, G, T) for any nucleotide sites, and multiple hits are unavoidable when divergence is old. Consequently, the high homoplasy leads to long-branch attraction [31], [45]. With haplotypes as character states, the potential number of states has no limit, and the chance of ‘multiple hits’ at a haplotype level is remote. Second, the genes as characters approach provides a convenient way to detect incongruence and to apply weighting schemes. With genomic data, data quantity is no longer an issue; conveniently determining incongruence and properly weighting the high quality data are becoming not only possible but also necessary [30], [46]. We weighted all genes equally in this study; this scheme was aimed to scale back the impact of highly variable genes, which produce many informative characters in a concatenated analysis but are often considered less reliable [47]. Several indices have recently been proposed to measure gene reliability, such as gene stationarity [46], gene informativeness [48], and internode certainty [30]. They can potentially be used to establish weighting schemes. Third, using gene as the unit of evolutionary studies makes better biological sense. What constitutes a character in evolutionary biology is a contentious issue [49], but gene is arguably the unit of inheritance and the unit of function. These traits make it a better choice as a unit of evolution than nucleotide site or amino-acid residue. One major criticism of analyzing each dataset (gene) separately is the loss of information when converting datasets into gene trees (see [43] for a review). With all branch length information being preserved in the conversion, however, this loss is minimum. Perhaps more importantly, a minimum loss of information is significantly outweighed by the aforementioned benefits with large genomic data.

At the operational level, the genes as characters approach also offers several advantages. First, selecting a best-fit model for each individual gene is relatively easy, and it is difficult to fit a realistic model for complex and heterogeneous data [41], [47]. Second, this approach can largely avoid the problems associated with computation time. Existing models and algorithms are challenged when applied to genome-wide combined phylogenetic analysis [32], [33]. Parsimony analysis, however, is computationally inexpensive, and has potential to handle large dataset. Lastly, with large dataset, errors are unavoidable. Our weighting scheme can effectively minimize the impact of several types of errors. During our analysis, we found some gene trees having one or more unusually long branches, and they were likely results from low quality alignments, non-orthologous genes, or potential assembling errors. Our weighting scheme is capable of making these gene trees almost uninformative by scaling their branches, and effectively weeds out these errors.

Although consensus tree and supertree approaches have been widely implemented [37], [41], [43], [50], exploring their use with genomic data is just beginning [51]. With the gene incongruence problem being at the center of phylogenomic analysis [30], [41], [44], [52], separated analysis of individual gene appears to be a necessary first step. How to summarize the individual gene trees into species tree, however, still need more investigation [51], [52]. Several approaches have been proposed, including consensus trees, minimizing deep coalescent events, and likelihood or Bayesian based probabilistic estimates of species tree methods [52]. More recently, Liu et al. [51] proposed a new coalescence based approach and compared various nonparametric and parametric methods. Salichos and Rokas [30], on the other hand, emphasized on selecting more reliable genes for phylogenetic reconstruction. We subscribe to a Popperian view on hypotheses regarding historical events. History is unknown, and the best-corroborated hypothesis represents the best guess with the available data [41], [53]. In this regard, parsimony analysis provides a computationally easy and philosophically sound approach in phylogenomics.

### From phylo- to genomics

Not only genomic data can improve the phylogenetic resolution of the tree of life, phylogenetic analysis can make significant contributions to the understanding of genomics as well. Phylogenies have always been the foundation for comparative studies of many aspects of genomes, such as genome size [54]. Additionally, phylogenetic analysis has a unique opportunity to contribute to genomics. Eisen (1998) early on suggested phylogenetic analysis as a tool to improve functional predictions for uncharacterized genes in a genome [55]. Recently, Lee et al. went further and suggested that genes or gene clusters that changed at a particular node could be used to derive or test hypotheses regarding the biological processes that are potentially responsible for the more interesting organismal level evolution [56]. In this regard, genes that are in conflict with the species tree may be the most interesting. Other aspects, such as modular evolution of genomes as in the case of the study of the origin of life [57], can also be explored in a phylogenetic context.

We have found clear evidence that the evolution of turtles was accompanied by positive selection on many genes (Table 2). On one hand, these genes may cloud the evolutionary history of turtles and make phylogenetic reconstruction difficult. In the case of turtles, genes that have experienced positive selection have a higher tendency to support the archosaur hypothesis (Table 2), which may contribute to the confusion regarding the phylogenetic position of turtles. On the other hand, they may offer important clues with respect to how turtles evolved, as Lee et al. suggested [56]. Along this line, detailed analysis with more genomic data may reveal genes that are responsible for the novel turtle body plan, which is a hot topic in modern evolutionary biology [58].

We also found clear evidence for modular evolution (Table 3). Genes in the same function category tend to support the same hypothesis. Although our study on turtles is not as pronounced as prokaryote to eukaryote dichotomy [57], it is not surprising that genes evolve in modular fashion. With the rapid accumulation of genomic data, genome wide evaluation of gene interactions and functional modules is an attractive direction for future research [59].

The genes as characters approach offers convenient tools for evaluating character evolution. In our case, we classified the genes into “supporting the archosaur hypothesis”, “supporting the lepidosaur hypothesis” and others with simplified nodes. In a more complex situation, genes can potentially be classified using several well-characterized indices, such as consistency index (CI) or retention index (RI). For example, a gene (character) has a CI greater than 0.9 on a particular topology (hypothesis) may be considered as “supporting the hypothesis”.

### Conclusions

Using genes as characters, our analysis of 4500+ genes suggested that the phylogenetic placement of turtles within vertebrates remains uncertain. Gene heterogeneity and hence conflicting information from various genes is likely the cause of this uncertainty. Although different data treatment may point to one way or another, a confident solution may be elusive.

The genes as characters approach may lessen systematic error associated with phylogenomic analysis. It may also offer convenience in substitution model selection and computation. Perhaps more importantly, it provides us with an opportunity to simultaneously understand the evolutionary history of individual genes as well as the whole genome. Recent advances in genomics will undoubtedly expedite the final resolution of the tree of life. In return, phylogenetics has much to offer for a better understanding of genomics.

## Materials and Methods

### Data acquisition

We first acquired high quality transcriptome data of a turtle species by RNA sequencing. Two adult Chinese pond turtles (*M. reevesii*) were purchased from a pet store at Chengdu, China. The specimens were euthanized by an overdose injection of sodium pentobarbital, and dissected immediately after death. RNA was separately extracted from brain, muscle, liver and heart tissues and mixed in approximately equal quantities. A cDNA library was constructed and subsequently sequenced on an Illumina HiSeq2000 platform; both were carried out by BGI Inc (Shenzhen, China). A total of 5G of raw read data were obtained.

A *de novo* assembly was performed. The raw reads were first cleaned by filtering out the adapter sequences. Reads with more than 5% unknown base calls, low quality reads (<Q20), and possible human and *Escherichia coli* contamination were also discarded. We used a multiple K-mer length and coverage cutoff value approach for assembly [60]. Thirty raw assemblies were produced using the program Velvet and OASES [61], [62] with five K-mer lengths (21, 31, 41, 51, and 61) and six coverage cutoff values (2, 3, 6, 10, 15, and 20). Sequence overlaps and redundancies were then eliminated to produce the final assembly using the programs CD-HIT-EST and CAP3 [63], [64]. All reads were mapped back to the final assembly to revisit all potential assembling errors using program SOAP2 [65].

Transcriptomic or genomic data of another ten vertebrate species were acquired from online databases. We first acquired genomic data of six vertebrate species from the Amniota Orthology Dataset (http://www.deep-phylogeny.org/hamstr/download/datasets/hmmer3/), which include green anole (*Anolis carolinensis*), chicken (*Gallus gallus*), human (*Homo sapiens*), opossum (*Monodelphis domestica*), zebra finch (*Taeniopygia guttata*), and western clawed frog (*Xenopus tropicalis*). Additionally, we downloaded transcriptomic data of the soft-shelled turtle (*Pelodiscus sinensis*), Nile crocodile (*Crocodylus niloticus*), royal python (*Python regius*), and tuatara (*Sphenodon punctatus*) [1], [66]–[68]. After adding the Chinese pond turtle, our dataset included a total of 11 species, and had at least two representatives of each major lineage of living amniotes (with the exception of a single crocodilian). The western clawed frog served as outgroup for phylogenetic analysis.

Putative orthologous genes were identified using the program HaMSTR (Hidden Markov Model based Search for Orthologs using Reciprocity; [36]) and the Amniota Orthology Dataset. For each core ortholog in the Amniota Orthology Dataset, HaMSTR searches a set of “unigenes” and identifies the best putative orthologs. The default parameters were used in all searches.

### Phylogenetic analysis

Phylogenetic trees were constructed based on both amino-acid and nucleotide sequences. Coding regions of the cDNA sequences were extracted and the UTRs were removed using local BlastX based on the amino-acid sequences. During this process, a small portion of coding sequences received low hit values of BlastX (e-value cut-off of 1e-5), and genes associated with these sequences were excluded from the subsequent analyses. Sequences of 1^st^ and 2^nd^ codon positions were also extracted for subsequent analyses.

Two datasets were constructed for phylogenetic analysis based on the levels of detail of the acquired data. Seven species (human, opossum, zebra finch, chicken, green anole, Chinese pond turtle, and western clawed frog) had many more orthologous genes than the other species, and formed a 7-species dataset. The second dataset, the 11-species dataset, had more taxa but fewer genes. These two datasets would allow us to test the effect of sampling, more genes or more taxa, and the 11-species dataset would also provide opportunity to test the crocodilian hypothesis.

#### Alignment

For amino-acid sequences, a newly developed fast and high-quality protein alignment method, Clustal Omega [69], was employed with its default parameters. All nucleotide sequences were aligned using a perl script with guidance from the amino-acid sequence alignment. Alignments of a random sample of genes were also manually checked to ensure the performance of the program. Poorly aligned columns and gap-rich regions were removed using TrimAl 1.2 [70] with the heuristic automated method.

#### Gene tree construction

We first analyzed each gene separately to obtain gene trees. Gene trees were constructed using maximum likelihood (ML) method with RAxML7.3.2 [71]. The JTT-F+G model for amino-acid sequences and GTR+G model for nucleotide sequences were used for all genes as a compromise to simplify the analysis process. Gene trees with unusually long branches (>1.5), which might have caused by low quality alignments, non-orthologous genes, or potential assembling errors, were excluded from subsequent analyses.

#### Concatenated data analysis

After removing dubious orthologs, the remaining amino-acid and nucleotide sequences were pooled together, respectively. The 7-species data (both amino-acid and nucleotide sequences) were analyzed using maximum parsimony (MP) and ML methods. For the 11-species data, the amino-acid sequences were analyzed using MP and ML methods, and the nucleotide sequences were analyzed using MP, ML and Bayesian inference. A separate MP analysis was also applied to the 11-species nucleotide data after removing all 3^rd^ codon position sequences.

The parsimony analysis was carried out using PAUP 4.10b [72]. Searching parameters included TBR branch swapping and 100 random addition replicates. A non-parametric bootstrap [73] with 1000 replicates was used to assess nodal support. For the large 7-species concatenated dataset, the data exceeded the memory limit of our PC (8G) and subsequently, all constant sites were removed prior to tree search. This would not impact the searching process but might have a small impact on bootstrap values [74].

Likelihood analysis was performed using RAxML. The JTT-F+G model for amino-acid sequences was selected as the best-fit model by a perl script (ProteinModelSelection.pl) implemented in the program. We used the GTR+G model for nucleotide sequences, which was the most commonly used DNA substitution model in RAxML. Nodal support was assessed by a non-parametric bootstrap with 100 replicates for amino-acid sequences and 1000 replicates for nucleotide sequences.

Bayesian inference was performed using PhyloBayes 3.3f with the CAT model [75], [76]. The model is a site-specific infinite mixture model, which is particularly suitable for phylogenomic studies with a large number of genes. We only applied this analysis to nucleotides of the 11-species dataset, because the 7-species dataset was too large to run this program (memory limited). We also removed all constant sites to improve the mixing as recommended by the authors. CAT-GTR model was used and two independent chains were executed. Trees were sampled every 10 cycles, and the first 20% of sampled trees were discarded as burn-in. The maximum difference and minimum effective size between the two independent runs were used to test the convergence; when the maximum discrepancy was <0.3 and minimum effective size was >50, the results were considered acceptable. The automatic stopping rule was employed; searches automatically stopped when the two chains reached convergence.

#### Genes as characters approach

Following the arguments of Doyle [35], each gene was treated as a single character. We used two methods to summarize the information from gene trees into species trees. First, we used a simple count method. The numbers of genes that support various hypotheses were counted and compared. The hypothesis supported by the highest number of genes was preferred. Second, we performed a more elaborated parsimony analysis following a systematic procedure (Figure 2).

Each gene was treated as a single ordered multi-state character. Ordered multi-state characters have high information content in their transformation series and are often considered “ideal” characters [77]. We assumed that these genes evolved largely independently to satisfy the independent character assumption of parsimony analysis.

Haplotypes of a gene were treated as character states and the gene trees were treated as the character-state trees. Step matrices were used to describe the character-state trees. The pairwise distance between two haplotypes on a gene tree represents the cost in tree-length units of the corresponding transformations on the species tree.

We weighted all characters (genes) equally. The maximum pairwise distance in each step matrix was set to ten and all other steps were proportionally scaled. Two digits after the decimal points were kept in the step matrices in order to force the use of floating-point evaluation of tree lengths rather than integer lengths. An in-house perl script was used to batch convert the ML gene trees into step matrix formats.

Finally, parsimony analyses were performed in PAUP. Heuristic search was employed with TBR branch swapping and 100 random addition replicates. Bootstrap values were estimated with 1000 replicates. The procedure is summarized in a flowchart and presented in Figure 2.

### Detecting incongruence

It is well recognized that data heterogeneity is a major problem in phylogenomic analysis [29], [30]. Different genes may have experienced different evolutionary histories and support different topologies. We examined the incongruence among genes with two methods.

#### SplitsTree analysis

We used a SplitsTree analysis to identify where, on the species tree, most incongruence occurred among the gene trees. All gene trees produced from separated analyses of genes were fed into the program SplitsTree 4 [37]. Consensus networks were constructed from those gene trees with split threshold values ranging from 0.15 to 0.30. The hyper-dimensional boxes (loops) in the networks represent areas with incompatible splits. The degree of denseness of boxes in a network reflects the intensity of contradictory evidence for grouping certain taxa, and the length of an edge is determined by the weight assigned to it [37]. In contrast, a network without boxes represents complete congruence.

#### Phylcon analysis

This analysis was used to detect different levels of support of various genes to a particular topology. Three rooted topologies of the 7-species data and four rooted topologies of the 11-species data were examined, which represented the primary alternative hypotheses. RAxML was used to compute the per-site log likelihood values for these topologies, with JTT-F+G (amino-acid) or GTR+G (nucleotide) substitution model. An AU test [78] was performed with CONSEL [79] based on these likelihood values. For any gene, a small *P* value (e.g., <0.05) for a topology indicates that the topology is significantly worse than the best tree, hence the gene rejects the topology. The *P* values for each individual gene and each topology were converted to heatmaps using the Phylcon package in R [38], which could be easily visualized.

### Exploring gene (character) evolution

Within a phylogenetic framework, we explored two aspects of gene evolution. It is well known that positive selection has a major impact on the evolutionary history of genes and may mislead phylogenetic construction [80], [81]. In addition, genes may evolve in a modular fashion. Genes with a similar function may co-evolve and support a particular tree topology [57].

#### Test of positive selection

We employed a maximum likelihood approach [39] to test for positive selection using the branch-site models implemented in the program CODEML [82]. This test detects positive selection on a particular tree branch and requires unrooted trees. Individual gene trees from RAxML were converted to unrooted trees. The turtle lineage was defined as foreground (ω_2_), and the other lineages were defined as background branches (ω_1_). The initial kappa and omega were set to 2.0 and 0.4, respectively. A Chi-square test was then conducted for each gene to determine whether ω_2_ was significantly higher than ω_1_ and ω_0_ (average ω across the entire tree), in which case the gene might have experienced positive selection. We conducted this part of the analysis for both the 7-species data and the 11-species data.

After identifying genes with positive selection, we performed a Chi-square test to examine any association between genes under selection and two primary alternative hypotheses, the archosaur and the lepidosaur hypotheses. This analysis was only applied to the 7-species data, which included a large number of genes and had high statistical power.

#### Gene functions and metabolic pathways

To assess gene modular evolution, we tested whether genes supporting a particular topology were over-represented in a particular function category or metabolic pathway. The Gene Ontology (GO) annotation and the Kyoto Encyclopedia of Genes and Genomes (KEGG) were used to classify gene function and we performed both analyses using BLAST2GO with an E cut-off value of 1e-6 [83]. We then conducted Chi-square tests to detect any relationship between gene functions and the archosaur and lepidosaur hypotheses. Only GO categories that had more than five genes were included in the Chi-square tests. This analysis was only applied to the 7-species data for high statistical power.

### Ethics Statement

All animal collection and utility protocols were approved by the Chengdu Institute of Biology Animal Use Ethics Committee.

## Supporting Information

Figure S1 **Length distribution of transcripts of the pond turtle (** ***Mauremys reevesii*** **).**
(EPS)

Table S1 **Summary of the transcriptome data for the pond turtle (*Mauremys reevesii*).**
(DOCX)
