# Supplementary material for: Using Genes as Characters and a Parsimony Analysis to Explore the Phylogenetic Position of Turtles
Source: PLoS One. 2013 Nov 21;8(11):e79348. doi: 10.1371/journal.pone.0079348 (PMC3836853; doi:10.1371/journal.pone.0079348)
Supplement: Table S1 — Summary of the transcriptome data for the pond turtle (Mauremys reevesii). (DOCX) [file pone.0079348.s002.docx]

Table S1. Summary of the transcriptome data for the Chinese pond turtle (*Chinemys reevesii*). Bp=base pairs

| *Chinemys reevesii* | |
| --- | --- |
| Total number of reads | 70,679,548 |
| Total length of reads (bp) | 6,371,959,320 |
| Read length (bp) | 90 |
| Total length of assembly (bp) | 66,311,060 |
| Number of transcripts | 61,810 |
| N50 length of transcripts (bp) | 1,571 |
| Mean length of transcripts (bp) | 1,072 |
| Maximum length (bp) | 19,374 |
